# Machine Learning Quantitation of Cardiovascular and Cerebrovascular Disease: A Systematic Review of Clinical Applications

**DOI:** 10.3390/diagnostics11030551

**Published:** 2021-03-19

**Authors:** Chris Boyd, Greg Brown, Timothy Kleinig, Joseph Dawson, Mark D. McDonnell, Mark Jenkinson, Eva Bezak

**Affiliations:** 1 Allied Health and Human Performance, University of South Australia, SA 5000, Australia; greg.brown2@unisa.edu.au; 2 South Australia Medical Imaging, Adelaide, SA 5000, Australia; 3 Department of Neurology, Royal Adelaide Hospital, Adelaide, SA 5000, Australia; timothy.kleinig@sa.gov.au; 4 Adelaide Medical School, The University of Adelaide, Adelaide, SA 5000, Australia; 5 Discipline of Surgery, University of Adelaide, Adelaide, SA 5000, Australia; joseph.dawson@adelaide.edu.au; 6 Department of Vascular & Endovascular Surgery, Royal Adelaide Hospital, Adelaide, SA 5000, Australia; 7 Computational Learning Systems Laboratory, UniSA STEM, University of South Australia, Mawson Lakes, SA 5095, Australia; mark.mcdonnell@unisa.edu.au; 8 Oxford Centre for Functional MRI of the Brain (FMRIB), Wellcome Centre for Integrative Neuroimaging, Nuffield Department of Clinical Neurosciences, University of Oxford, Oxford OX3 9DU, UK; mark.jenkinson@ndcn.ox.ac.uk; 9 Cancer Research Institute, University of South Australia, Adelaide, SA 5001, Australia; eva.bezak@unisa.edu.au; 10 Department of Physics, University of Adelaide, Adelaide, SA 5000, Australia

**Keywords:** artificial intelligence, machine learning, cta, vascular disease

## Abstract

Research into machine learning (ML) for clinical vascular analysis, such as those useful for stroke and coronary artery disease, varies greatly between imaging modalities and vascular regions. Limited accessibility to large diverse patient imaging datasets, as well as a lack of transparency in specific methods, are obstacles to further development. This paper reviews the current status of quantitative vascular ML, identifying advantages and disadvantages common to all imaging modalities. Literature from the past 8 years was systematically collected from MEDLINE^®^ and Scopus database searches in January 2021. Papers satisfying all search criteria, including a minimum of 50 patients, were further analysed and extracted of relevant data, for a total of 47 publications. Current ML image segmentation, disease risk prediction, and pathology quantitation methods have shown sensitivities and specificities over 70%, compared to expert manual analysis or invasive quantitation. Despite this, inconsistencies in methodology and the reporting of results have prevented inter-model comparison, impeding the identification of approaches with the greatest potential. The clinical potential of this technology has been well demonstrated in Computed Tomography of coronary artery disease, but remains practically limited in other modalities and body regions, particularly due to a lack of routine invasive reference measurements and patient datasets.

## 1. Introduction

As the first and second leading causes of global mortality, ischemic heart disease and stroke demonstrate the need for improved tools in the management of occlusive vascular disease [1]. In spite of the global incidence of both being on the decline, regional trends vary, and the total number of persons affected continues to rise due to population growth [2,3]. Patients with cardiovascular disease leading to stroke and myocardial infarction often require significant medical imaging in the acute, sub-acute, and chronic settings, using a range of imaging modalities. Vascular imaging is then used as a key source of information in the determination of appropriate clinical management, from a range of potential pharmacological and surgical approaches [4,5,6]. The large datasets obtained from this imaging are traditionally interpreted qualitatively by clinicians and are highly heterogeneous, varying due to differences in patient, imaging technology, and site scanning protocols.

Artificial Intelligence (AI) is a broad field usually characterised by two key commonalities; the design of machines to mimic human cognition, and the design of machines to complete a task whilst optimising the outcome [7]. The potential applications of machine learning (ML) to medical imaging is an emergent field, attracting growing research investment [8]. Patients suspected of cardiovascular and neurovascular diseases undergo multiple medical imaging procedures, which generates large amounts of data used in conjunction with conventional medical datasets such as patient records. Whilst these records are meticulously maintained for each individual patient, pooling this data into multi-site collaborative databases will bolster the development of ML tools and automated ML analysis. Within medicine, ML has emerged as a prominent approach for automated diagnosis and image segmentation; for detailed background of ML theory and associated medical applications, readers are directed to [9,10].

Selecting the appropriate algorithm from a great many possibilities, with each having inherent strengths and weaknesses, is a core component in development of any ML technology. Discussion of algorithm selection, technical explanation of algorithm function, or explanation of supporting mathematics are beyond the scope of this work. ML, as the currently preferred approach for analysing medical imaging datasets, refers to algorithms used to build a model capable of identifying correlations between data features. The correlations in data features are identified by “seen” input data, before then being applied to previously unseen data to perform predictions.

This seen input data can be provided in two forms, supervised and unsupervised, with each using different classes of algorithms for model development. Supervised machine learning uses input data labelled by a relevant domain expert, such as a medical specialist. These labels may be in the form of sematic segmentation, labelling at a voxel level by contouring structures from an image, or classify at an image level with labels singularly classifying the image as a whole. These labels could identify the presence of pathology in an image, or sort images based on disease stage or sub-type [10]. Common supervised ML algorithms include Support Vector Machines (SVM), k-nearest neighbour, deep neural networks, and random forest [11]. In unsupervised machine learning algorithms such as fuzzy C-means, experts do not provide data labels, instead allowing the algorithm to determine classifications independently [9,10]. Although medical imaging data can be acquired using a variety of modalities or protocols and is notoriously heterogeneous, datasets can be considered as decimal arrays with two or more dimensions. Each pixel or voxel then represents levels of grey between 0 and the maximum bit-depth of the image. These matrices of grey level values are the input data which ML algorithms use for their model development.

The appearance, volume, and variability of data made available to researchers, as well as the clinical condition being investigated, all determine which machine learning algorithm and imaging modality are employed. Invasive Coronary Angiography (ICA), Computed Tomography Angiography (CTA), two-dimensional ltrasound (US), Intravascular ultraSound (IVUS), Magnetic Resonance Imaging (MRI), Optical Coherence Tomography (OCT), Invasive Coronary Angiography (ICA), and Nuclear Medicine (NM) each play a role in diagnostic and therapeutic vascular imaging, in the process generating datasets suitable for ML analysis.

Previous reviews of medical imaging ML applications have provided a broad inspection of literature across all diseases [12,13,14] and covered narrow topics in great detail, such as a single imaging modality [15,16], task [17], vascular location (overwhelmingly cardiac vessels [18,19,20,21,22]), or have covered a specific combination of all three [23,24,25].

Imaging of vasculature following myocardial infarction or stroke is routinely performed using a range of imaging techniques, as each provides unique information. The currently published research in clinically useful vascular ML demonstrates different stages of development, varying with both vascular region and imaging modality. For example, US and IVUS ML research has a wealth of carotid publications, but a comparative dearth of coronary papers. Conversely, coronary CTA products have been commercialised for routine clinical use, whilst preliminary studies into non-coronary CTA products are lacking or absent. Both cardiovascular and neurovascular diseases have large disease burdens, especially in developed countries [26], but current ML research is not proportionate, and coronary applications are far more advanced. This is due to many factors, including coronary disease being the global leading cause of death and recent advances in the hardware and software used for coronary CTA, improving both image quality and acquisition speed. These techniques have the potential to assist in clinical management and reduce disease burdens, but the highly heterogeneous state of current literature makes the identification of future areas difficult. Review, synthesis, and critical analysis of ML approaches from a wide range of modalities and vascular locations is needed to identify commonalities and gaps in research. With this, researchers are better placed to ensure future work focuses on relevant clinical needs and more effectively translate into clinical practice, improving patient care.

This work reviews the current status of combined knowledge for ML based quantitation of both coronary and neurological vascular disease. The current effectiveness of ML to provide quantitative descriptors of vessel disease will be examined, as well as the impact of this quantitation on clinical decision making and patient outcomes, for a wide range of medical imaging modalities.

## 2. Materials and Methods

A systematic review was conducted in accordance with guidance included in the Preferred Reporting Items for Systematic Reviews and Meta-Analyses (PRISMA) statement [27].

### 2.1. Data Sources

A computerised literature search of Ovid MEDLINE and Elsevier SCOPUS was performed to find full text, original articles published in the eight years prior to 29 January 2021 in medicine, engineering, or computing, which investigated ML analysis of human vascular imaging. The search terms were combined as follows: (ALL (“Machine Learning” OR “Neural Network” OR “Support Vector” OR “Random Forest” OR “Bayesian Network” OR “Nearest Neighbor”)) AND (ALL (plaque OR calcification OR ulceration OR stenosis))) AND (ALL (quantif* OR quantitati*))) AND NOT (ALL (spectroscop* OR alzheimer OR “tau” OR “amyloid” OR “lung” OR “multiple sclerosis”)).

### 2.2. Data Extraction and Quality Assessment

Studies satisfying these criteria underwent assessment of title and abstract by two authors (C.B and E.B), with equivocal papers additionally reviewed by a third (G.B). This included analysis of abstract and titles which indicated quantitative machine learning analysis of human vascular imaging, with pertinent studies continuing to full text review. Using a standardised data extraction form, relevant information was collected, including (a) participant characteristics (patient numbers, patient age, age range, and sex), (b) data characteristics (number of images, imaging modality, and disease site), (c) AI characteristics (algorithm, algorithm parameters, quantitation results, and model validation/cross-validation), and (d) per patient outcomes (gold standard, sensitivity/specificity/accuracy, and Area Under the Curve (AUC)).

## 3. Results

### 3.1. Literature Search

**Figure 1:**
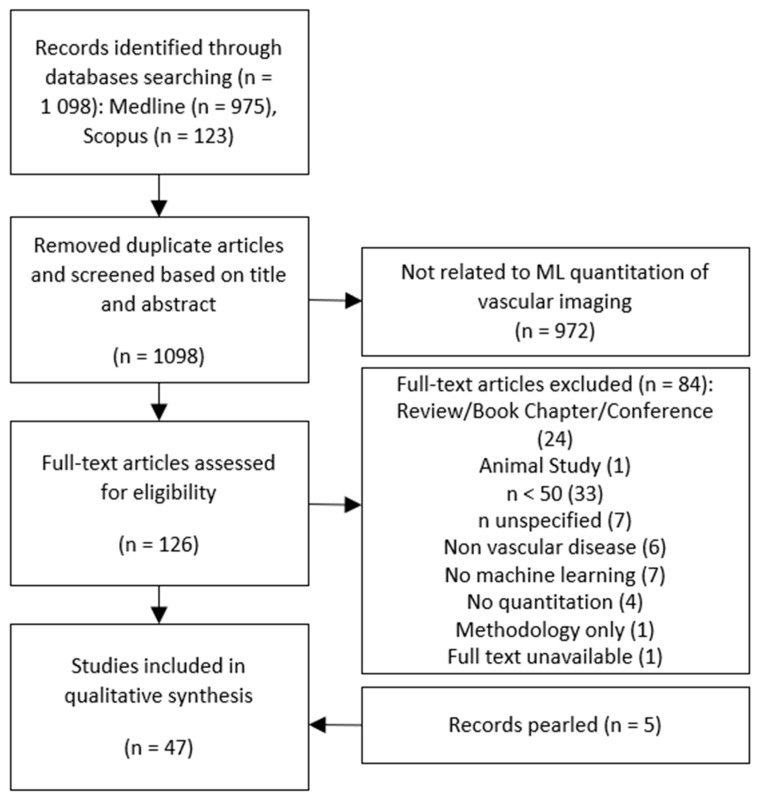
PRISMA (Preferred Reporting Items for Systematic Reviews and Meta-Analyses) flow diagram of literature selection process.

The detailed literature selection process is shown in Figure 1. The systematic search identified 1098 articles, with most records deemed outside the area of interest based on title and abstract review. Although several studies quoted large datasets, some were referencing total number of pixels [28], Region-Of-Interest (ROI) subsets extracted from small image numbers [29], or number of images extracted from a small number of patients [30,31,32,33]. To manage any potential bias and avoid inclusion of overfit or low variability datasets, articles where total unique patient numbers were less than or equal to 50 were also excluded. Seven articles failed to identify the number of patient datasets used and were also excluded. The reference lists of the remaining 42 papers were then pearled, and an additional 5 papers were included, for a total of 47.

As previously mentioned, the appropriate ML techniques depend on both the type and quantity of available data, as well as the ML researchers’ experience, skill, and currency of knowledge. Experienced ML researchers consider these and other practical factors in determining the most suitable algorithm, although algorithm choice is not unique, and several algorithms may be suitable for one data type, or multiple data types may be analysed successfully with one algorithm. The literature identified was sorted by imaging modality and algorithm selection, before extracting and tabulating data relevant for the development of an integrative overview of automated ML vascular quantitation. Vascular pathology quantification was most often performed using CTA (27) and ultrasound (6) imaging, as well as several publications using other modalities (9). All but one IVUS study [34] was excluded due to insufficient patient numbers [28,35,36,37,38,39,40,41], highlighting the need for more large-scale research in this modality. A few studies attempting vascular quantitation from Nuclear Magnetic Resonance (NMR) [42] and Nuclear Medicine (NM) [43,44,45] imaging were also included, as well as one publication using ICA [46].

### 3.2. Modality Specific Vascular Imaging

#### 3.2.1. Computed Tomography Angiography

CT for vascular imaging has increased in utility due to consistent improvement in CT technology and is now the most prevalent imaging modality for quantitative ML analysis, spurred on by recent research into the low diagnostic yield of ICA [47]. Technological advancements allowing increased tube rotation speeds, reducing motion blur, smaller detector element sizes increasing spatial resolution, and the clinical implementation of advanced filtration and reconstruction techniques, have all improved visualisation of vasculature. A summary of CTA quantitative analysis is shown in Table 1.

ML vascular analysis from CTA imaging is a well-established field [48,49], with assessment of ML and CTA-based calculation of Fractional Flow Reserve (FFR) published by multiple sites [50,51,52,53,54,55,56,57] and now explored by several comprehensive reviews [58,59,60]. FFR is defined as the ratio of blood flow, proximally and distally to a coronary vascular lesion, measured under pharmacologically maximised coronary blood flow [61]. Han et al. [62] performed CT-based FFR quantification using resting perfusion CT, instead of the more widely used CTA, obtaining sensitivity, specificity, and accuracy values shown in Table 2.

Siemens^®^ Syngo cFFR (Erlangen, Germany) software was widely used across the literature, in various development stages, for the quantitation of FFR, and results are summarised in Table 3. This technology, based on coronary deep neural network research published previously [63], was used by both [53] and Yu et al. [57] to quantify FFR as part of a wider evaluation of lesion specific ischemia and functional significance. Both authors found a limited but measurable benefit of including CT-based FFR in plaque analysis. The resulting Area Under the Curve (AUC) of the Receiver Operating Characteristic (ROC) measured by Coenen et al. [64], Hu et al. [51], and Yu et al. [54] for CT-based FFR were all similar to that quoted above, with all using invasive FFR measurements as a benchmark. FFR clinical significance was typically based on a threshold of 0.8 and equivocality around this value (termed the ‘grey zone’ [57]) was observed for all measurement techniques, although to varying degrees [54].

Various other aspects of vascular health were quantitatively analysed using machine learning, based on CT imaging datasets. In these instances, hold-out [65,66,67,68] and k-fold cross validation [50,65,67], two ML validation techniques, were employed to improve algorithm performance, with both used in conjunction where possible to minimise bias. Quantification of individual plaque risk and Major Adverse Cardiovascular Events (MACE) risk stratification was examined in several papers, both with [53,69] and without [65,67,70] CTA-derived FFR.

Specific data properties used in ML analysis, often termed ‘features’, contribute unequally to the overall model performance and the inclusion of features should be done judiciously [71]. In vascular analysis, features such as remodelling index, plaque length, mean lumen diameter, mean luminal area, and napkin ring sign showed some utility as discriminating features, whilst others such as calcified plaque burden and spotty calcification had near zero information gain, meaning they were unlikely to provide any predictive power to model performance [50,53,69,70,72]. Several equivocal image features were identified as both strongly and weakly predictive by different publications, and these included total plaque volume, non-calcified plaque volume, and Agatston score (a score based on the maximal Hounsfield Unit (HU) value observed in CT imaging of coronary artery calcifications) [50,53,69,72]. This equivocality suggests feature selection should be performed systematically for each research work individually, due to variabilities in available data and project outcomes. Generally, ranking of features by information gain was consistent for CTA imaging, with quantitative image parameters contributing greater to model function than qualitative imaging parameters or clinical/laboratory values [71]. The automated and semi-automated determination of Agatston score has also been investigated separately [68,73,74,75,76,77], although some publications utilise thresholding and individual/direct image analysis approaches, rather than ML.

Although useful, plaque quantitation is not the only informative quantitative metric in cardiovascular risk management. Motwani et al. [78], with access to one of the largest CTA imaging datasets in current vascular ML research, compared existing coronary risk stratification quantifiers such as Agatston score and Framingham risk score with a ML LogitBoost model for quantification of five-year All-Cause Mortality (ACM). Analysis of 44 imaging features and 25 clinical features found a statistically significant increase in the AUC of all-cause mortality risk, compared to Framingham risk score alone (0.79 v 0.61), although no validation cohort was investigated. Similar ML performance was observed in the ACM risk quantification of an 86,155 strong cohort of Korean patients [79], with the AUC values of ML and Agatston score mortality risk of 0.82 and 0.70, respectively. Despite the large dataset, performance in a validation cohort was not found to be statistically significant (AUC: 0.78 v 0.62), possibly suggestive of model overfitting in spite of large sample sizes.

Zreik et al. [80] analysed clinical plaque significance using a convolutional neural network developed in-house, investigating only left ventricular myocardium CT images with clusters of pixels belonging to myocardium identified by fast K-means clustering. This method contrasted to the direct vessel imaging approached employed widely throughout the literature across all modalities. The model development utilised 50% random dropout and 10-fold cross validation to minimise overfitting, and plaque significance was benchmarked against invasive FFR. Multiple FFR thresholds were investigated for their impact on the determination of individual lesion clinical significance, ranging from 0.72 to 0.8. Technical parameters were similarly varied, using multiple fast K-means cluster values (1–1000) for division of the myocardium and several convolutional auto-encoders. The mean AUC across the 50 cross-validations was 0.74 ± 0.02, with the majority of algorithm and clinical setting combinations returning AUC values between 0.65 and 0.75. In a continuation of this work, van Hamersvelt et al. [81] applied the analysis method developed previously to intermediary stenosis (defined as 25–69% stenosis as assessed by invasive coronary angiography), noting improved sensitivity and AUC with a slight decrease in specificity.

ML-based coronary vessel FFR quantitation using CTA datasets is rapidly becoming established as a clinical methodology, although as with all clinical practices, critical evaluation remains ongoing. Two elements of FFR quantitation subject to this critical evaluation were the significance of X-ray tube peak kilovoltage (kVp) dependencies undertaken by De Geer et al. [82], and the role of partial volume effects by Freiman et al. [83]. De Geer et al. [82] concluded no impact in FFR quantitation between 100 and 120 kVp and slightly better agreement between ML and invasive FFR quantitation at 100 kVp. The large pixels of CT imaging, in comparison to ultrasound and fluoroscopy, can result in partial volume effects in which assigned pixel values represent a mean of values from multiple structures contained within. Consideration of the role these partial volume effects play in FFR were found to improve both the specificity (0.51 to 0.73) and AUC (0.76 to 0.8) of FFR ML quantitation as compared with angiographically determined values.

Work in quantitative machine learning cerebrovascular analysis was extremely limited. No clear practical or technical reason could be found to sufficiently explain why research in this area is so sparse, particularly given the comparative examination throughput and vessel lumen diameter of coronary vessels and many cerebral vessels of clinical significance. The absence of a routinely performed invasive quantitation comparison methods and no collaborative multi-centre databases appear to be two possible reasons, with just two groups attempting to investigate this area [66,84]. The work of Park et al. [66] investigated aneurysm detection by expert operator from CT imaging, with and without the assistance of a segmentation deep neural network. An incremental but statistically significant increase in reader sensitivity, accuracy, and agreement was found. Deep learning approaches to neurological CTA were noted by the authors as absent from current literature prior to their investigation, suggesting future development may provide clinically useful results warranting further research. A preliminary pilot study by Acharya et al. [84] into carotid lumen segmentation and pathology quantification from CT data produced sensitivities, specificities, and accuracies of 0.88, 0.865, and 0.902, respectively, using an SVM classifier with radial basis functions. Statistically significant high-level features (*p* < 0.01) were identified for the differentiation of symptomatic and asymptomatic plaques, in particular higher energy and lower entropy in symptomatic images due to increased texture complexity. The generalisation of these results was limited, however, as only 20 consecutively sampled patients were used.

#### 3.2.2. Ultrasound

The excellent soft tissue resolution and sub-millimetre spatial resolution of ultrasound is well suited to imaging of vascular detail, readily applicable to the large, superficial, and accessible common carotid artery bifurcation. As shown in Table 4 in contrast with CTA investigations, cardiac studies were comparatively few, with external ultrasound ill-suited to coronary vasculature investigation. Study sample sizes often included less than 50 patients and were too small for generalisation, particularly for highly invasive IVUS studies [28,36,39,40], although recently published works have attempted to address this [34].

Research examining segmentation of vascular anatomy and pathology using ultrasound is extensive, with publications reaching as back as far as 2000 [85,86], although advancements enabling automated quantification are comparatively few. The development of ML tools capable of identifying at-risk asymptomatic carotid disease and providing decision support is the primary focus of current ultrasound research. Bae et al. [34] developed and compared the performance of multiple ML methods for identification of vulnerable coronary plaques from IVUS investigations, as defined by the presence of a fibrous thin cap atheroma. Results were compared with OCT, the highest resolution vascular imaging currently clinically available. The implementation of tools such as these would support the management of neurovascular and cardiovascular diseases, although to date ultrasound is not widely employed for initial diagnosis in acute stroke [87,88,89].

As expected from the acquisition technology employed, US examinations offered an alternative set of image parameters compared to CTA, with some having utility as machine learning features. The most consistent of these features was the measurement of carotid Intima Media Thickness (IMT).

IMT is below the spatial resolution of CTA and cannot be visualized, but has been correlated to increased vascular risk [90]. This makes US a uniquely practical tool for measurement of IMT thickness. Segmentation of IMT has been performed using several US imaging parameters such as the Hough transform [91], frequency domain analysis [92], and pixel intensity analysis, the latter using a deep learning single-layer feed-forward neural network. This method achieved a sensitivity and specificity greater than 97% (shown in Table 5) when compared to expert manual segmentation [33].

Another analysis utilising image features unique to ultrasound was evaluation of the discrete Fréchet distances of greyscale cumulative distribution functions, as compared to idealised functions, from Huang et al. [93]. This greyscale distribution analysis for individual plaques in combination with a k-nearest neighbour classification system sorted plaques into echo-rich, intermediate, and echo-lucent, with association between echo type and plaque vulnerability established elsewhere [94,95].

The correlation of echogenicity and carotid plaque vulnerability was also utilised by Pedro et al. [96], Roy-Cardinal et al. [97] and Golemati et al. [98], although the former used a simplistic ROC cut-off analysis to produce a semi-quantitative plaque vulnerability indicator. Echogenicity was used in conjunction with both general greyscale image features such as Rayleigh parameters or grey level co-occurrence matrix (GLCM) decomposition and features more specific to ultrasound imaging such as homodyned-K parametric mapping, wavelet energy decomposition, or elastography. The two other papers, however, provided comprehensive examination of both symptomatic and asymptomatic carotid stenosis, detailing machine learning techniques, patient descriptors, and clinical outcomes. Using a range of extracted image parameters, US elastography, and plaque motion synchronisation, the composition and clinical significance of symptomatic and asymptomatic plaques were analysed with a random forest classifier. Roy-Cardinal et al. [97] also compared US plaque to composition determined by MRI, as well as comparing patient symptomology to experimental predictions, keeping results focused on improvement of clinical patient outcomes, the ultimate target of any ML model.

#### 3.2.3. Other Imaging Modalities

Whilst US and CTA are the dominant imaging modalities, several other methods exist which are useful in the diagnosis and management of vascular disease, including MRI, NM, OCT, and ICA. Application of these modalities showed the greatest variability with ML approaches used on both direct image analysis and routinely obtained clinical descriptors. Image analysis research from these modalities used markedly smaller sample sizes, especially when compared to US and CT patient databases described above. Some reasons may include increased invasiveness and fewer centres to facilitate multi-institutional research or cost, although the exact reason remains unclear. ICA showed extensive use as a gold standard for all other imaging modalities, but ML image analysis of ICA was limited. Table 6 summarises ML vascular quantitation results performed using modalities other than CT and ultrasound.

##### Magnetic Resonance Imaging

Only two identified MRI publications satisfied all selection criteria. Waddle et al. [99], applied an SVM model with a fitcsvm function and radial bias function kernel to MRI, Magnetic Resonance Angiography (MRA), and functional MRI (fMRI) data of moyamoya patients. The hemispheric blood flow of patients was compared with healthy controls, and ML classification of hemispheres was performed with a resulting sensitivity, specificity, and AUC of 0.7, 0.83, and 0.71, respectively. The authors commented that ML techniques are notably underutilised in vascular imaging, despite finding their results “collectively offer increased support that both anatomical and functional hemodynamic imaging can serve as important machine learning inputs” [99].

Wu et al. [100] developed a deep convolutional neural network to investigate MRA of carotid vessels, applied to blood signal suppressed, or “black blood” images. Patient imaging was supplied from two previously collected research datasets [101,102] with carotid segmentation performed and quantitatively compared to expert manual segmentation. In addition to analysis of individual patient slices, data from the images immediately before and after each slice were considered, producing a “2.5-dimension” dataset. Carotid lesion types, as defined by the American Heart Association [103], were determined from segmented vessels and identified as atherosclerotic or non-atherosclerotic, with maximum ML accuracy and AUC of 0.89 and 0.95, respectively, in relation to expert decisions.

Four other publications utilising magnetic resonance techniques for quantitative vasculature analysis were identified, which although not satisfying inclusion criteria, warrant further discussion. These publications show future potential of MRI both for direct analysis of vessels, and the possible application of ML to information unique to MRI such as relaxation times or spectroscopic analysis.

The relationship between carotid vessel image parameters and stroke risk was investigated by Van Den Bouwhuijsen et al. [104] using logistic regression, from a large, pre-existing patient database [105]. Despite the simplicity of the approach, this method supports the importance of large datasets, associating stroke risk with intraplaque haemorrhage, carotid wall thickness, and calcification. Automated segmentation of Carotid artery plaque directly from contrast enhanced MRI has also shown promise, with a recent study [106] using 35 patients to obtain automated segmentations with a Dice score and true-positive of 0.89 and 0.93, respectively, as compared to manual analysis.

Van Hespen et al. [107] used sub-millimetre isotropic voxels to image ex vivo circle of Willis specimens and train a convolutional neural net to measure wall thicknesses for intracranial aneurysm. Although the results were promising, with less than 0.1 mm error in intracranial vessel wall estimation, a three patient validation sample size, and long acquisitions in a high field MRI (7T) places limits on any immediate clinical translation.

Forssen et al. [42] demonstrated the breadth of MRI to obtain clinically useful information when combined with machine learning, utilising supervised ML to quantify 256 metabolites associated with coronary artery disease, through Nuclear Magnetic Resonance spectroscopy.

##### Nuclear Medicine

The large difference in spatial resolution between CTA, US, and IVUS discussed previously is similar when comparing CTA and many common NM procedures. Instead of imaging vessels directly, Nakajima et al. [43,44,45] used approximately 2000 Technetium-99m Myocardial Perfusion Imaging (MPI) studies from Swedish and Japanese datasets to train an artificial neural network in the detection of perfusion defects and ischemia. Each model was tested on an in-house cohort of 106 patients and compared to both expert readers and a >50% stenosis gold standards, with version 1.1 of the model further validated on 364 patients. As shown in Table 7, improvement was observed in the upgraded 1.1 version ML tool, with sensitivities and specificities in excess of 87% for all patients, peaking at 88 and 100%, respectively, in patients with no history of prior infarction or coronary revascularization.

Quantitative and semi-quantitative descriptors routinely obtained during cardiac Positron Emission Tomography (PET) were also analysed using six ML algorithms and linear regression. Obstructive coronary disease status was determined from a sample of 88 patients, with performance statistics approaching 0.9 and SVM performing best.

## 4. Discussion

Across many imaging modalities and organ vasculatures, clinically useful information can be gained by quantitation of atherosclerotic disease and associated infarction. The task of segmenting and quantifying vascular pathology is currently both repetitive and laborious, as well as requiring specialist expertise. This combination makes the task well suited to automation by machine learning.

The ultimate focus of any research into ML vascular quantitation across all modalities and vascular territories must remain the meaningful and positive impact to patient outcomes. Although ML analysis of coronary CTA imaging has progressed furthest towards broad clinical use, accuracies in the range of 70–80% show that research remains to be done. Researchers should be buoyed by these results however, as they do demonstrate the clinical potential of quantitative vascular ML steadily becoming actualised.

### 4.1. Limitations and Future Work

The works reviewed in this paper achieved performance comparable to current methods and in some cases demonstrated commercialised ML vascular analysis in clinic. Despite the success of these methods, several hurdles remain before ML vascular image quantitation is ready to be applied to patient care in some contexts.

#### 4.1.1. Common Machine Learning Limitations

Limited data availability and a lack of code accessibility (the black box reputation of ML) are limitations seen in many machine learning applications, within medicine and beyond. Although not unique to quantitative vascular machine learning, both are nonetheless important considerations in any future research.

Data management is the foundation from which all ML research is undertaken, and the comparative infancy of most current vascular ML quantitation research provides the ideal opportunity to establish standardised data sharing and result reporting approaches, which will support the development of useful clinical technology into the future. Construction of vascular imaging benchmark datasets has begun to address both the problem of transparency and data availability simultaneously. Unfortunately, current datasets are not truly publicly available (instead limited to ethically approved research trials with data available to a select group of researchers) and participant numbers insufficient in most cases for generalisation to the diversity of patients seen in many hospitals. These small datasets offer developers a starting point for useful evaluation and comparison of privately developed models, on the understanding that this database is not also used for model training. Some identified datasets include the MACHINE consortium [64], CONFIRM registry [108], and PARADIGM [109] CTA datasets, as well as the annual challenge databases of the Medical Image Computing and Computer Assisted Intervention (MICCAI) society from all modalities, all of which have been used multiple times throughout the literature [67,73,79,83,110,111]. The handling of patient imaging for use in medical machine learning is a complex issue being contended with around the world [112]. Only once a broad framework of requisite protections is in place to allow ethical data handling can large databases be constructed to allow robust model development.

Many algorithms produced by current research have been developed and evaluated using patient data that is not independently accessible and which cannot be externally validated for reproducibility. Once these models are demonstrated by researchers to provide benefit, some are then packaged into commercial offerings with little or no public details on further changes or advances [63]. Without an external or open access reference database, further research benchmarking of the performance of comparative models in a transparent and useful fashion is impossible. This issue of data availability was discussed in both Coenen et al. [64] and Cho et al. [46], in which the authors stated patient data will not be provided for the purpose of independent result validation, with no explicit justification given. Failure by ML developers to make data or model design specifics available perpetuates the black box stereotype of these tools and adversely impacts clinician confidence when considering whether to use potentially beneficial tools. Gao et al. [113] attempted to address this with the provision of detailed algorithm processes in a summarised step-by-step fashion that, although not equivalent to open access, is an interesting intermediary step to improving reproducibility. Waddle et al. [99] was the only publication identified in this review which actively supported reproducibility, explicitly stating a provision for de-identified data to be made available on request.

For CTA studies, and in particular for the assessment of FFR, the commercial availability of deep learning platforms, such as cFFR v1.0–3.0 (Siemens Healthineers, Erlangen, Germany), CAAS vFFR (Pie-Medical, Maastricht, The Netherlands), and HeartFlow^®^ FFR-CT (HeartFlow, Redwood City, CA, USA) limited the provision of algorithm details due to commercial and intellectual property interests.

#### 4.1.2. Reference Standards

The standardisation of cardiac analysis from CT imaging by the American Heart Foundation [114] has provided consistent segmentation nomenclature in this region. Similarly, the definition of FFR methodology [61] allowed results of works investigating FFR using CTA imaging to be compared with this standard metric [70,115]. Routine quantification of FFR for coronary CTA provides the ideal reference standard for ML based quantification from CTA imaging. Well defined coronary vascular segments, a defined methodology for FFR determination and the inclusion of quantitative invasive vascular quantitation for every patient allows method performances to be compared and approaches to be benchmarked. The routine quantitation of vascular disease in carotid vessels is currently limited to manually measured criteria such as that outlined by NASCET [116]. Although such measurements are clinically useful [117], the risk of inter and intra observer variability cannot be discounted, particularly when incorporated into large multi-nation, multi-centre databases, the likes of which will be required for robust machine learning model development.

Although not a singular reference measurement, the development and adoption of MICCAI and AAPM publications providing detailed methodologies for standardised evaluation of algorithms examining stenosis and lumen segmentation or coronary vascular quantitation supports model evaluation and comparison in a similar way [76,118,119]. Research into model benchmarking has begun, but is still in the early stages, with small patient sample sizes (*n* =10) and limited algorithms investigated (*n* = 4) [120].

#### 4.1.3. Image Standards

Beyond the absence of invasive quantitation as a reference standard, the variation in acquisition methodology between sites also presents a challenge for generalisation from vascular machine learning. CT image reconstruction methods (filtered back projection, iterative reconstruction, and AI enhanced reconstruction), MRI settings (bandwidth, TR and TE times, matrix size), training of sonographers, and combinations of administered dose and acquisition time in nuclear medicine, all vary with institution. Image appearance preferences also vary between institutions and even reporting clinicians within one institution, with acquisition parameters changed accordingly. Such wide variability necessitates greater robustness in any clinical models to account for application of developed technologies to image appearances not previously encountered.

#### 4.1.4. Reporting Standards

Reporting of machine learning results throughout the literature was vague and inconsistent for all modalities. Statistical metrics varied widely with combinations of sensitivity, specificity, accuracy, and AUC reported inconsistently, and compared to different gold standards. The nature of ML development and analysis further complicated inter-publication comparison with the result section of each paper quoting sensitivity, specificity, and AUC values obtained using different image feature combinations, algorithm parameter settings (such as SVM kernel), and repeating this process for multiple algorithms, resulting in large tables of statistics encompassing a wide range of values. Finally, the reporting of studies in vascular quantitation allowed results to be reported on a per-patient, per vessel, per vessel segment or per lesion basis, with some papers reporting results for several of these.

## 5. Conclusions

The review highlights that open access data, or a systematic and independent validation solution, are essential to the continued research growth of vascular ML quantitation. This issue was identified and best explained by Zreik et al. [121], who despite having access to a large imaging research facility, observed that *“[with] a sufficiently large and diverse data set, a deeper CNN-only…analyzing a large single volume along the artery, could be employed to perform the presented analyses. However, obtaining such a large data set remains highly challenging…”*. Neural networks, and convolutional neural networks especially, have been widely recognised across multiple modalities as the most promising candidates for vascular imaging ML analysis. Despite this, other articles identified by this review [99] concluded, similarly to Zreik et al. [121], that current data is insufficient for clinical CNN implementation. Although many techniques exist for maximising model performance from limited data, large quantities of unique datasets are essential to clinical model performance [122,123]. The imaging data accessible to vascular ML researchers at present is forcing the selection of algorithms to be heavily influenced by available datasets, not necessarily those that are the best performing or most informative. Once standardised datasets are made readily available, transparent and standardised reporting of results will promote collaboration and improve both the development of ML techniques and the clinical confidence in the use of the technology.

## Figures and Tables

**Table 1:** Computed Tomography Angiography (CTA) characteristics.

Organ	ML Prediction Endpoint	Author (Year)	No. of Patients (M/F)	ML Approach or Software	ML Validation	Gold Standard	Contrast Used?
Brain	Faster clinician identificiation of intracranial aneurysm	Park (2019)	662 (157/505)	3-D CNN.	75/14/11 train/validation/test	Clinicians’ segmentation (*n* = 8)	Yes
Heart	5 year ACM	Motwani (2017)	10,030 (5628/4402)	LogitBoost	k-fold cross validation (*n* = 10)	Existing clinical or cCTA metrics	Yes
	ACM	Han (2019)	86,155 (59,745/26,410)	LogitBoost	70/30 holdout validation	Mortality status at follow up (median 4.6 years)	No
	CAD—Calcium scoring	Shahzad (2013)	366 (280/86)	k-Nearest Neighbour	57/43 holdout validation	Expert Calcium Scoring	No
	Coronary vessel centreline extraction	Wolterink (2019)	82	3-D CNN. + SVM	MICCAI 2008 CAT08 dataset—32 pre segmented cCTA images	Clinicians’ segmentation (*n* = 3)	Yes
	FFR variation with kVp	De Geer (2019)	351	Syngo^TM^ cFFR	12,000 virtual coronary models	Invasive coronary angiography FFR	Yes
	Functional stenosis significance	Coenen (2018)	351 (258/93)	Syngo^TM^ cFFR	12,000 virtual coronary models	Invasive coronary angiography FFR	Yes
		Hae (2018)	1132 (860/272)	Light Gradient Boosting Machine	83/17 Holdout validation k-fold cross validation (*n* = 3)	79 external patients CAAS-5 software	Yes
		Han (2018)	252 (178/74)	SmartHeart Software	Leave one out cross validation	Invasive coronary angiography FFR	Yes
		Kurata (2019)	74 (56/18)	Syngo^TM^ cFFR	12,000 virtual coronary models	Invasive coronary angiography FFR	Yes
		van Hamersvelt (2019)	126 (97/29)	As described in Zreik (2018)	k-fold cross validation (*n* = 50)	Invasive coronary angiography FFR	Yes
		Zreik (2018)	166 (128/38)	3-D CNN + SVM	Manual segmentation of 40 patients	Invasive coronary angiography FFR	Yes
		Dey (2018)	254 (162/92)	Ensemble classification approach (Supervised ensemble learning)	k-fold cross validation (*n* = 10)	Invasive coronary angiography FFR	Yes
		von Knebel Doeberitz (2018)	84 (54/30)	Syngo^TM^ cFFR	12,000 virtual coronary models	Invasive coronary angiography FFR	Yes
		Wardziak (2019)	90 (61/29)	Syngo^TM^ cFFR	12,000 virtual coronary models	Invasive coronary angiography FFR	Yes
		Yu (2018)	129	Syngo^TM^ cFFR	12,000 virtual coronary models	Invasive coronary angiography FFR	Yes
		Yu (2019)	180	Syngo^TM^ cFFR	12,000 virtual coronary models	Invasive coronary angiography FFR	Yes
		Hu (2018)	105 (73/32)	Syngo^TM^ cFFR	12,000 virtual coronary models	Invasive coronary angiography FFR	Yes
		Nous (2019)	351 (258/93)	Syngo^TM^ cFFR	12,000 virtual coronary models	Invasive coronary angiography FFR	Yes
		Zreik (2019)	163 (126/37)	Recurrent CNN	50/10/40 train/validation/test	Clinicians’ segmentation (*n* = 2)	Yes
	Functional stenosis significance (myocardial bridging)	Zhou (2019)	161 (103/58)	Syngo^TM^ cFFR	12,000 virtual coronary models	41 control patients Clinicians segmentations (*n* = 2)	Yes
	MACE related lesions	Tesche (2016)	92 (57/35)	Syngo^TM^ Coronary Plaque Analysis 2.0.3		Invasive coronary angiography FFR	Yes
		von Knebel Doeberitz (2019)	82 (51/30)	Syngo^TM^ cFFR	12,000 virtual coronary models	Invasive coronary angiography FFR	Yes
	Machine learning ischemia risk score	Kwan (2020)	352 (238/114)	Ensemble classification approach (Supervised ensemble learning)	k-fold cross validation (*n* = 10)	Invasive coronary angiography FFR	Yes
	Plaque based risk stratification	Priyatharshini (2017)	76	Active contour model-based region growing		Agatston score	Yes
	Plaque based risk stratification	Zhang (2019)	129	Dense U-net	k-fold cross validation (*n* = 5) 40 “*orScore”* database patients	2 expert Agatston score	No
	Plaque based risk stratification	van Rosendael (2018)	8844 (5102/3742)	Gradient boosted decision trees	80/20 holdout validation k-fold cross validation (*n* = 5)	Clinician segmentation + cCTA risk score	Yes
	Plaque based risk stratification	Wang (2019)	530	3D-Resnet deep neural network	56/17/27 train/validation/test	Agatston Score	No
	Plaque stability	Al’Aref (2020)	468	XGBoost	80/20 holdout validation k-fold cross validation (*n* = 10)	Invasive coronary angiography	Yes
	Rapidly progressing plaque	Han (2020)	1083 (624/459)	LogitBoost, Naïve Bayes, BayesNet, AdaBoost, Random Forest, Bagging, Stacking, MLP, Sequential Minimimal Optimization, ADTree	70/30 holdout validation	Atherosclerotic cardiovascular disease risk score/duke coronary artery disease score	Yes

CNN = Convolutional Neural Network, ACM = All-cause mortality, cCTA = Coronary computed tomography angiography, CAD = Coronary artery disease, FFR = Fractional flow reserve, kVp = Kilovoltage peak, cFFR = Software computation of FFR, SVM = Support vector machine, MACE = Major adverse cardiovascular events, MLP = Multilayer perceptron, ADTree = Alternating decision tree.

**Table 2:** Model performance statistics for machine learning (ML) quantitation using CTA.

Organ	ML PredictionEndpoint	Statistics Quoted	Author (Year)	Sample Size	Sensitivity		Specificity		Accuracy		AUC	
					Min	Max	Min	Max	Min	Max	Min	Max
Brain	Faster aneurysm identification	Per clinician	Park (2019)	818 exams		0.89		0.98		0.93		
Heart	5-year ACM	Per patient	Motwani (2017)	10,030 patients							0.79	0.79
	ACM (CAD)	Per patient	Han (2019)	86,155 patients							0.74	0.78
	CAD—Calcium scoring	Per patient	Shahzad (2013)	366 patients		0.84						
	Coronary vessel centreline extraction		Wolterink (2019)	82 patients								
	FFR variation with kVp	Per vessel	De Geer (2019)	525 vessels	0.74	1.00	0.73	0.79	0.77	0.86	0.82	0.90
	Functional stenosis significance	Per patient	Coenen (2018)	525 lesions	0.82	0.96	0.60	0.83	0.75	0.91		
			Hae (2018)	1132 lesions	0.73	0.84	0.76	0.85	0.74	0.84	0.80	0.91
			Han (2018)	252 patients	0.52	0.71	0.61	0.85	0.64	0.68		
			Kurata (2019)	91 lesions	0.33	0.90	0.38	0.91	0.59	0.85		
			van Hamersvelt (2019)	126 patients		0.85		0.48		0.72		0.76
			Zreik (2018)	166 patients		0.70		0.71		0.71	0.62	0.85
		Per lesion	Dey (2018)	2758 artery segments								0.84
			von Knebel Doeberitz (2018)	103 lesions	0.62	0.88	0.33	0.68			0.61	0.93
			Wardziak (2019)	96 lesions		0.76		0.72		0.74		0.84
			Yu (2018)	166 lesions							0.85	0.88
			Yu (2019)	208 lesions	0.81	0.94	0.82	0.87	0.83	0.86	0.87	0.94
		Per vessel	Hu (2018)	117 lesions	0.61		0.91		0.82		0.86	0.92
			Nous (2019)	525 arteries	0.79	0.88	0.72	0.80	0.75	0.83	0.82	0.88
		Per segment	Zreik (2019)	676 lesions							0.62	0.80
	Functional stenosis significance (myocardial bridging)	Per lesion	Zhou (2019)	161 patients							0.65	0.77
	MACE	Per patient	Tesche (2016)	258 lesions	0.63	0.83	0.73	0.83			0.72	0.82
		Per lesion	von Knebel Doeberitz (2019)	82 patients		0.82		0.79				0.94
	Machine learning ischemia risk score	Per vessel	Kwan (2020)	352 patients								0.78
	Plaque based risk stratification	Per patient	Priyatharshini (2017)	76 patients						0.91		
		Per lesion	Zhang (2019)	129 patients	0.86	0.91						
		Per vessel	van Rosendael (2018)	8844 patients								0.77
			Wang (2019)	530 patients								
	Plaque stability	Per lesion	Al’Aref (2020)	582 lesions								0.77
	Rapidly progressing plaque	Per patient	Han (2020)	1083 patients							0.79	0.83

ACM = All-cause mortality, CAD = Coronary artery disease, FFR = Fractional flow reserve, kVp = Kilovoltage peak, MACE = Major adverse cardiovascular event.

**Table 3:** Model performance statistics using Siemens Syngo cFFR.

Organ	ML Prediction Endpoint	Statistics Quoted	Author (Year)	Sample Size	Sensitivity		Specificity		Accuracy		AUC	
					Min	Max	Min	Max	Min	Max	Min	Max
Heart	FFR variation with kVp	Per vessel	De Geer (2019)	525 vessels	0.74	1.00	0.73	0.79	0.77	0.86	0.82	0.90
	Functional stenosis significance	Per patient	Coenen (2018)	525 lesions	0.82	0.96	0.60	0.83	0.75	0.91		
			Kurata (2019)	91 lesions	0.33	0.90	0.38	0.91	0.59	0.85		
			von Knebel Doeberitz (2018)	103 lesions	0.62	0.88	0.33	0.68			0.61	0.93
			Wardziak (2019)	96 lesions		0.76		0.72		0.74		0.84
			Yu (2018)	166 lesions							0.85	0.88
			Yu (2019)	208 lesions	0.81	0.94	0.82	0.87	0.83	0.86	0.87	0.94
		Per vessel	Hu (2018)	117 lesions	0.61		0.91		0.82		0.86	0.92
			Nous (2019)	525 arteries	0.79	0.88	0.72	0.80	0.75	0.83	0.82	0.88
	Functional stenosis significance (myocardial bridging)	Per lesion	Zhou (2019)	161 patients							0.65	0.77
	MACE related lesions	Per lesion	von Knebel Doeberitz (2019)	82 patients		0.82		0.79				0.94

FFR = Fractional flow reserve, kVp = Kilovoltage peak, MACE = Major adverse cardiovascular events.

**Table 4:** Ultrasound characteristics.

Organ	ML Prediction Endpoint	Author (Year)	No. of Patients (M/F)	US Type	ML Approach	ML Validation	Gold Standard
Brain	Carotid elastography	Roy-Cardinal (2019)	66 (47/19)	B-Mode	Random forest	0.632+ validation	Patient symptoms
	Carotid plaque echomorphology	Golemati (2020)	77 (59/18)	B-Mode	Random forest	Leave one out	Clinicians’ segmentations (*n* = 1)
		Huang (2018)	153	B-Mode	k-nearest neighbours	k-fold cross validation (*n* = 3)	Grayscale median
		Pedro (2014)	109 (34/75)	B-Mode	Cutoff of ROC	Leave one out	Clinician assignment of symptomatic plaque status
	Carotid plaque segmentation	Menchon-Lara (2016)	67	B-Mode	Neural Network	66/33 Holdout validation	Clinicians’ segmentations (repeated) (*n* = 2)
	IMT measurement & plaque detection	Hassan (2013)	300	B-Mode	Fuzzy C-mean & probabilistic neural network		Clinicians’ segmentations (*n* = 1)
Heart	Probability of OCT identified thin-cap fibroatheroma	Bae (2019)	517 (382/135)	IVUS	ANN, SVM, naïve bayes	k-fold cross (*n* = 5) 80/20 Holdout	Presence of OCT thin-cap fibroatheroma

B-mode = Anatomical ultrasound, ROC = Receiver operating characteristic curve, IMT = Intima-media thickness, OCT = Optical coherence tomography, IVUS = Intravascular Ultrasound, ANN = Artificial neural network, SVM = Support vector machine.

**Table 5:** Model performance statistics for ML quantitation using B-mode US and IVUS.

Organ	ML Prediction Endpoint	Statistics Quoted	Author (Year)	Sample Size	Sensitivity		Specificity		Accuracy		AUC	
					Min	Max	Min	Max	Min	Max	Min	Max
Brain	Carotid elastography	Per patient	Roy-Cardinal (2019)	66 patients							0.79	0.83
	Carotid plaque echomorphology	Per lesion	Golemati (2020)	77 patients	0.69	0.86	0.68	0.88	0.69	0.85	0.79	0.90
		Per patient	Huang (2018)	315 frames	0.68	0.81	0.63	0.89	0.72	0.85	0.71	0.83
		Per image	Pedro (2014)	146 frames	0.66	0.70	0.76	0.80	0.73	0.77	0.79	0.89
	Carotid plaque segmentation		Menchon-Lara (2016)	67 patients								
	IMT measurement & plaque detection	Per patient	Hassan (2013)	300 frames		0.98		0.98		0.98		0.98
Heart	Probability of OCT identified thin-cap fibroatheroma	Per image	Bae (2019)	41,101 frames	0.81	0.84	0.61	0.79	0.76	0.82	0.74	0.82

IMT = Intima-media thickness, OCT = Optical coherence tomography.

**Table 6:** Characteristics of modalities other than CT and ultrasound.

Organ	ML Prediction Endpoint	Author (Year)	No. of Patients (M/F)	Imaging Modality	ML Approach	ML Validation	Gold Standard
Brain	Atherosclerosis identification	Wu (2019)	1482	MRI	2.5D CNN (U-Net)	90/10 holdout validation	Clinicians’ segmentations (n unknown)
	Cerebral blood flow & cerebrovascular reactivity	Waddle (2019)	53 (10/43)	MRI		LOO-CV k-fold cross validation (*n* = 3)	Invasive coronary angiography
Heart	Functional stenosis significance	Cho (2019)	1501 (1157/344)	Invasive Angiography	XGBoost	80/20 holdout validation k-fold cross validation (*n* = 5)	79 external patients
		Gao (2019)	0	Computer Generated CTA	Recurrent Neural Net		180 external patients w/Invasive coronary angiography FFR
	Presence of CAD	Forssen (2017)	3409	NMR quantification of 256 metabolites	Random Forest + Penalized Logistic Regression	k-fold logistic regression (*n* = 50)	Coronary angiography reports
	Probability of myocardial ischemia	Nakajima (2015)	106 (65/41)	NM (Tc-MPI)	Artificial Neural Net.		Clinicians’ segmentations (*n* = 3)
		Nakajima (2017)	1001 (751/250)	NM (Tc-MPI)	Artificial Neural Net.	364 (265/98) external patients	Clinicians’ segmentations (n unknown)
		Nakajima (2018)	106 (65/41)	NM (Tc-MPI)	Artificial Neural Net.		Clinicians’ segmentations (*n* = 3)
		Wang (2020)	88 (83/5)	PET (^13^N-NH_3_ & ^18^F-FDG)	SVM, Logistic Regression, Decision Tree, Linear Discriminant Analysis, Naïve Bayes, k-Nearest Neighbour, Random Forest	60/40 holdout validation	Invasive coronary angiography

MRI = Magnetic resonance imaging, CNN = Convolutional neural network, LOO-CV = Leave one out cross validation, CTA = Computed tomography angiography, CAD = Coronary artery disease, NMR = Nuclear magnetic resonance, NM = Nuclear Medicine, Tc-MPI = Technetium-99m myocardial perfusion imaging, PET = Positron emission tomography, ^13^N-NH_3_ = Nitrogen-13 ammonia, ^18^F-FDG = Fluorine-18 fluorodeoxyglucose, SVM = Support vector machine.

**Table 7:** Model performance statistics for ML quantitation using modalities other than CT and ultrasound.

Organ	ML Prediction Endpoint	Statistics Quoted	Author (Year)	Sample Size	Sensitivity		Specificity		Accuracy		AUC	
					Min	Max	Min	Max	Min	Max	Min	Max
Brain	Atherosclerosis identification		Wu (2019)	18,915 frames					0.81	0.89	0.87	0.95
	Cerebral blood flow and cerebrovascular reactivity		Waddle (2019)	112 hemispheres	0.43	0.7	0.67	0.83			0.65	0.71
Heart	Functional stenosis significance	Per patient	Cho(2019)	1501 frames	0.72	0.84	0.77	0.89	0.81	0.85	0.87	0.90
		Per patient	Gao (2019)	13,000 synthetic trees	0.84	0.92	0.75	0.89			0.89	0.94
	Presence of CAD	Per patient	Forssen (2017)	3409 patients	0.94	0.94	0.21	0.28	0.71	0.73	0.68	0.71
	Probability of myocardial ischemia	Per patient	Nakajima (2015)	106 patients		0.69		0.62		0.66	0.88	0.97
		Per patient	Nakajima (2017)	1001 patients							0.90	0.93
		Per patient	Nakajima (2018)	106 patients	0.78	0.87	0.96	0.98	0.89	0.92	0.89	0.96
		Per patient	Wang (2020)	159 vessels	0.72	0.91	0.32	0.84	0.65	0.81	0.62	0.86

CAD = Coronary artery disease.
